# A CT based radiomics analysis to predict the CN0 status of thyroid papillary carcinoma: a two- center study

**DOI:** 10.1186/s40644-024-00690-y

**Published:** 2024-05-15

**Authors:** Zongbao Li, Yifan Zhong, Yan Lv, Jianzhong Zheng, Yu Hu, Yanyan Yang, Yunxi Li, Meng Sun, Siqian Liu, Yan Guo, Mengchao Zhang, Le Zhou

**Affiliations:** 1 Department of Radiology, China-Japan Union Hospital of Jilin University, Changchun, 130000 China; 2 Department of Radiology, Affiliated Fifth People’s Hospital of Chengdu University of Traditional Chinese Medicine, Chengdu, 611130 China; 3 Department of Radiology, The People’s Hospital of Bao’an, Shenzhen University, Shenzhen, 518101 China; 4 Life Sciences, GE Healthcare, Shenyang, 110000 China; 5 Department of Thyroid Surgery, China-Japan Union Hospital of Jilin University, Changchun, 130000 China

**Keywords:** CN0, CT imaging, Radiomics, Papillary thyroid carcinoma

## Abstract

**Objectives:**

To develop and validate radiomics model based on computed tomography (CT) for preoperative prediction of CN0 status in patients with papillary thyroid carcinoma (PTC).

**Methods:**

A total of 548 pathologically confirmed LNs (243 non-metastatic and 305 metastatic) two distinct hospitals were retrospectively assessed. A total of 396 radiomics features were extracted from arterial-phase CT images, where the strongest features containing the most predictive potential were further selected using the least absolute shrinkage and selection operator (LASSO) regression method. Delong test was used to compare the AUC values of training set, test sets and cN0 group.

**Results:**

The Rad-score showed good discriminating performance with Area Under the ROC Curve (AUC) of 0.917(95% CI, 0.884 to 0.950), 0.892 (95% CI, 0.833 to 0.950) and 0.921 (95% CI, 868 to 0.973) in the training, internal validation cohort and external validation cohort, respectively. The test group of CN0 with a AUC of 0.892 (95% CI, 0.805 to 0.979). The accuracy was 85.4% (sensitivity = 81.3%; specificity = 88.9%) in the training cohort, 82.9% (sensitivity = 79.0%; specificity = 88.7%) in the internal validation cohort, 85.4% (sensitivity = 89.7%; specificity = 83.8%) in the external validation cohort, 86.7% (sensitivity = 83.8%; specificity = 91.3%) in the CN0 test group.The calibration curve demonstrated a significant Rad-score (P-value in H-L test > 0.05). The decision curve analysis indicated that the rad-score was clinically useful.

**Conclusions:**

Radiomics has shown great diagnostic potential to preoperatively predict the status of cN0 in PTC.

**Supplementary Information:**

The online version contains supplementary material available at 10.1186/s40644-024-00690-y.

## Key Points

**•** Radiomics has shown great diagnostic potential to preoperatively predict the status of cN0 in PTC.
**•** Retrospective two-center study showed that radiomics has provides greater diagnostic confidence.

## Introduction

Cervical LN postoperative metastasis has been associated with local recurrence and distant metastasis, negatively affecting patient survival [1–3]. Therapeutically, central neck LN dissection (CND) has been recommended for patients with lymph node involvement [4–6]. European Thyroid Association (ETA) and the Korean Society of Thyroid Radiology (KSThR) divides lymph nodes into three groups according to US features, including suspicious, indeterminate LNs and benign LNs. We defined indeterminate LNs and benign LNs as clinically node-negative (cN0). For clinically node-negative (cN0) patients, American Thyroid Association (ATA) and Asian guidelines are controversial on whether to perform prophylactic central neck dissection(pCND). However, we found that some lymph nodes diagnosed as cN0 confirmed by ultrasound were pathologically proved as metastatic lymph nodes. The risk of surgical complications such as hypoparathyroidism and recurrent laryngeal nerve injury may occur after performing pCND [3, 7–10]. There may be a risk of postoperative recurrence without pCND [11]. Whether to implement preventive central neck dissection (pCND) depends on the authenticity of cN0. Therefore, we need to find a way to improve the authenticity of cN0 state detection. As a non-invasive examination, imaging is an effective alternative examination method.

Traditional imaging plays a key role in the preoperative assessment of cervical LN metastasis for PTC patients. Preoperative neck ultrasound (US) for cervical LNs has been recommended for all patients undergoing thyroidectomy [2, 4–6]. In a meta-analysis, CT demonstrated a higher sensitivity of 62% than US (51%) and there was no difference in specificity in detecting LN metastasis, but the performance was still unsatisfactory [8, 12]. In summary, the limited sensitivity of traditional imaging for cervical LN metastasis may cause many patients to be misdiagnosed as cN0.

Radiomics is a method that extracts large amount of features from radiographic medical images using algorithms of data characterization, and made some positive result in preoperative evaluation of LN metastasis of variety of cancers [13–18]. Up to now, there is no relevant study about preoperatively predicting the authenticity of cN0 status by radiomics model based on CT images.

Therefore, the purpose of this study is to establish a radiomics model based on arterial CT images of LNs to predict LN status and explore if the model can be used for detecting cervical LN metastasis of clinical LN negative (cN0) patients in PTC.

## Materials and methods

### Patients/lymph node

Ethics license was received from the Science Ethics Committee. This study is a retrospective study and informed consents were waived. This work complied with the Declaration of Helsinki.

Patients were selected from a cohort who was consecutively treated in two independent centers. The study was conducted between January 2015 and February 2019 in Center #1, and from December 2016 to December 2019 in Center #2.

### Inclusion criteria

**Fig. 1:**
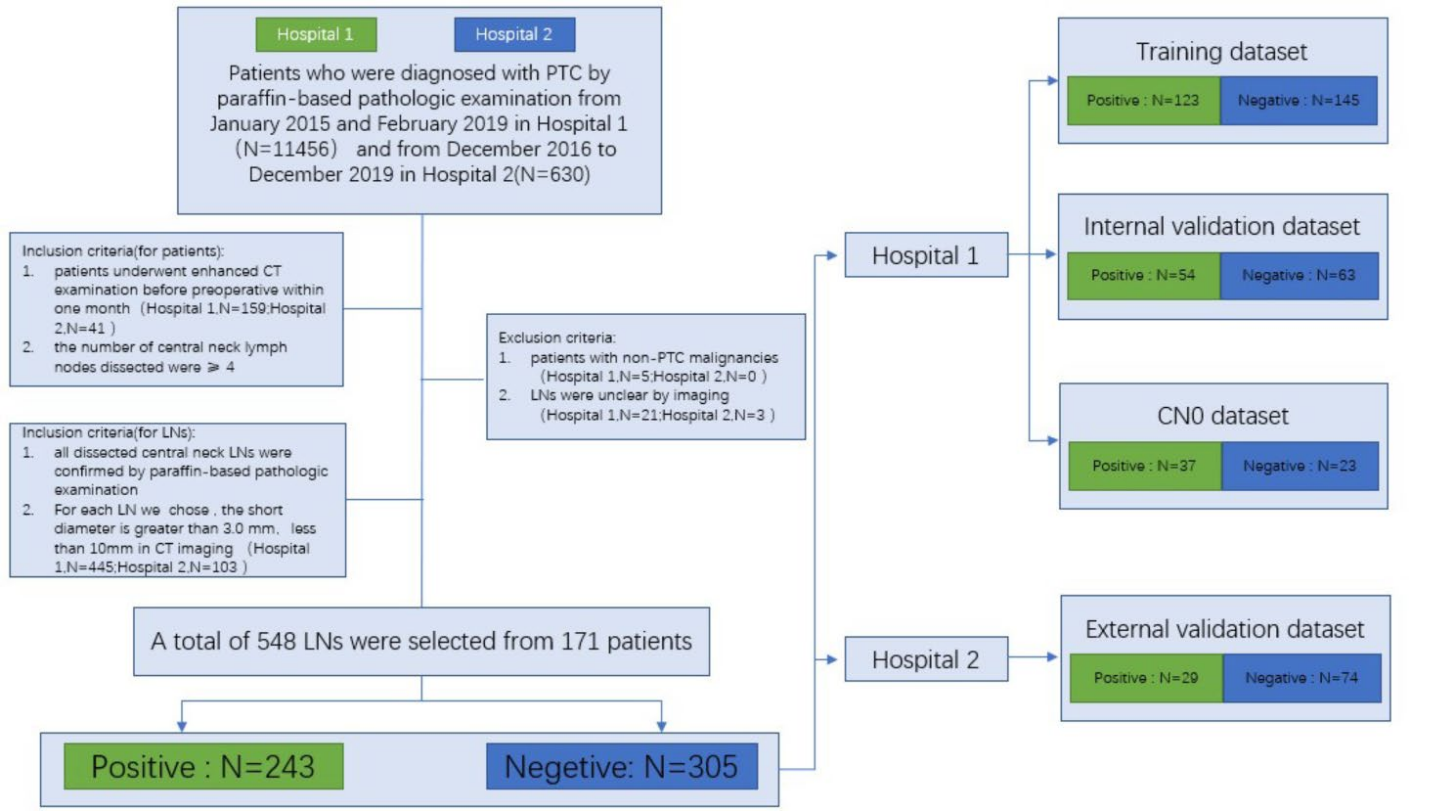
The workflow of LNs enrollment

Patients: a)patients who were diagnosed with PTC by paraffin-based pathologic examination and underwent enhanced CT examination before preoperative within one month; b)the number of central neck lymph nodes dissected were ≥ 4;

Lymph node: (a) all dissected central neck LNs were confirmed (i.e. metastasis/no metastasis) by paraffin-based pathologic examination; (b) the short diameter of the lymph node (for each selected LN) should be greater than 3.0 mm, less than 10 mm in CT imaging. c). For lymph nodes in the cN0 group, we selected the indeterminateand benign lymph node group according to the European Thyroid Association (ETA) and the Korean Society of Thyroid Radiology (KSThR) guidelines (Fig. 1).

### Study deign

In this study, thelymph nodes were from two centers: lymph nodesfrom center 1were divided into two cohorts according to the proportion of 7:3, one cohorts was used to established a radiomics prediction model we called radiomics score (Rad-score), one cohortswas used as an internal validation group; lymph nodesfrom center 2 were used as external validation group; Then the Rad-score is used to diagnose LN metastasis in cN0 goup. The CN0 group was additionally selected from Center 1.

### CT protocol

All patients underwent contrast-enhanced neck CT, within one month before operation. The details of the CT protocol are indicated in the supplemental data (Appendix 1).

### Radiomics analysis based on CT images

#### Image segmentation

Arterial phase CT images were retrieved from PACS and then exported to an open-source software (ITK-SNAP 3.8.0; www.itksnap.org) for segmentation. A total of 402 ROIs from were manually delineated into arterial phase CT images, by two independent readers, to evaluate the inter-observer reproducibility of the feature extraction. Three months after the initial segmentation, 50 ROIs from were selected randomly to be re-delineated by the same radiologist to analyze the intra-observer reproducibility of the feature extraction.

#### Radiomic feature extraction

A total of 396 radiomics features were automatically generated using AK software (Artificial Intelligence Kit, AK, GE Healthcare, China), which included a first order histogram, high order texture and morphological features (Supplemental Data, Appendix 2).

### Feature selection and Modeling

In order to ensure repeatability and robustness of the model, features with ICC > 0.75 in both inter- and intra-reader agreement assessments were considered stable and remained for subsequently analysis. The remaining radiomics features were normalized by z-score method, and the abnormal numbers were replaced by a median value. In order to reduce overfitting of the model, high-dimensions of features were pre-selected with Kruskal-Wallis test. Thereafter, the least absolute shrinkage and selection operator (LASSO) regression with 10-fold cross-validation was repeated by a hundred times to select the most useful predictive radiomics features with non-zero coefficient in the training cohort. Upon conclusion of these steps, a Rad-score was calculated for each LN by linear combination of the selected features that were weighted with their respective coefficients. Then, the radiomics score formula derived from the training set was applied to internal validation group (from center #1) and external validation group (from center #2).

### Performance evaluation in cN0 PTC patients

The performance of the Rad-score was tested in an independent validation cohort composed of cN0 PTC patients. We selected some lymph nodes with N1 and N0 by postoperative paraffin pathology, but whosepreoperative clinical stage was cN0in Center #1. All lymph nodes were identified on CT images by thyroid surgeons. All the central LNs of all patients were manually depicted as ROI by reader 1 according to the above mentioned criteria. Then extract the specified features of each ROI and use Rad-score for diagnosis LNs status.

### Statistical analysis

Statistical analysis was conducted with R software (version 3.4.1; http://www.Rproject.org). Statistical tests were 2-sided, and a P-value of < 0.05 indicated statistical significance. The names of the R packages used for analyses were as follow: ICC was calculated using “lme4” package. LASSO regression analysis was performed using the “glmnet” package. Multivariate logistic regression were performed using the “rms” package. ROC curves were plotted using the “pROC” package. Delong test was used to compare the AUC values of training set, test sets and cN0 group. Decision curve analysis was performed using the “dca. R” package. Calibration curve and Hosmer-Lemeshow test were conducted using the “ModelGood” package.

## Results

### Baseline characteristics

The process of patient recruitment is illustrate in Fig. 1. According to the inclusion criteria, 133 PTC patients (male, *n* = 41; female, n = 92) in Center #1 (China-Japan union hospital of Jilin University) and 38 PTC patients (male, n = 9; female, n = 29) in Center #2 (The People’s Hospital of Bao’an) were selected. The clinical characteristics of these patients are shown in Table 1, while the baseline characteristics of the total enrolled LNs (n = 548) are presented in Table 2.

**Table 1:** Patient characteristics in Hospital 1 and Hospital 2

	Hospital 1	Hospital 2	P value
**Age (Mean ± SD, years)**	43.02 ± 10.94	40.68 ± 10.91	0.247
**Sex, No. (%)**			0.393
Female	92 (69.2)	29 (76.3)	
Male	41(30.8)	9 (23.7)	
**Central LNM, No. (%)**			0.148
Negative	81 (60.9)	28 (73.7)	
Positive	52 (39.1)	10 (26.3)	

Abbreviations: LNM, lymph node metastasis

**Table 2:** Lymph node characteristics

	Training cohort (n=268)	Internal validation cohort (n=117)	External validation cohort (n=103)	CN0_Test cohort (n=60)	P value
**Age (Mean** ± **SD, years)**	42.83 ± 10.43	41.49 ± 11.48	41.06 ± 10.53	40.07 ± 8.57	0.19
**Sex, No (%)**					0.000
Female	174 (64.9)	82 (70.1)	67 (65.0)	18 (30)	
Male	94 (35.1)	35 (29.9)	36 (35.0)	42 (70)	
**Central LNM, No. (%)**					0.970
Negative	145 (54.1)	63 (53.8)	74 (71.8)	23 (38.3)	
Positive	123 (45.9)	54 (46.2)	29 (28.2)	37 (61.7)	

Abbreviations: LNM, lymph node metastasis; LN, lymph node

### Statistical analysis

Receiver operating characteristic (ROC) curves were plotted to evaluate the diagnostic performance of the radiomics score in both training and testing sets. Area under the ROC curve (AUC) with 95% confidence interval (CI), specificity, sensitivity, and accuracy were calculated. DeLong test was used to compare the differences of AUC values between different models in the training and testing set. To evaluate whether the models were well-calibrated or not, calibration curves were plotted in both training and testing sets. Decision curve analysis (DCA) was conducted to determine the clinical usefulness of the models by quantifying the net benefits at different threshold probabilities in both training and testing sets.

### Development of the Radiomics Model

**Fig. 2:**
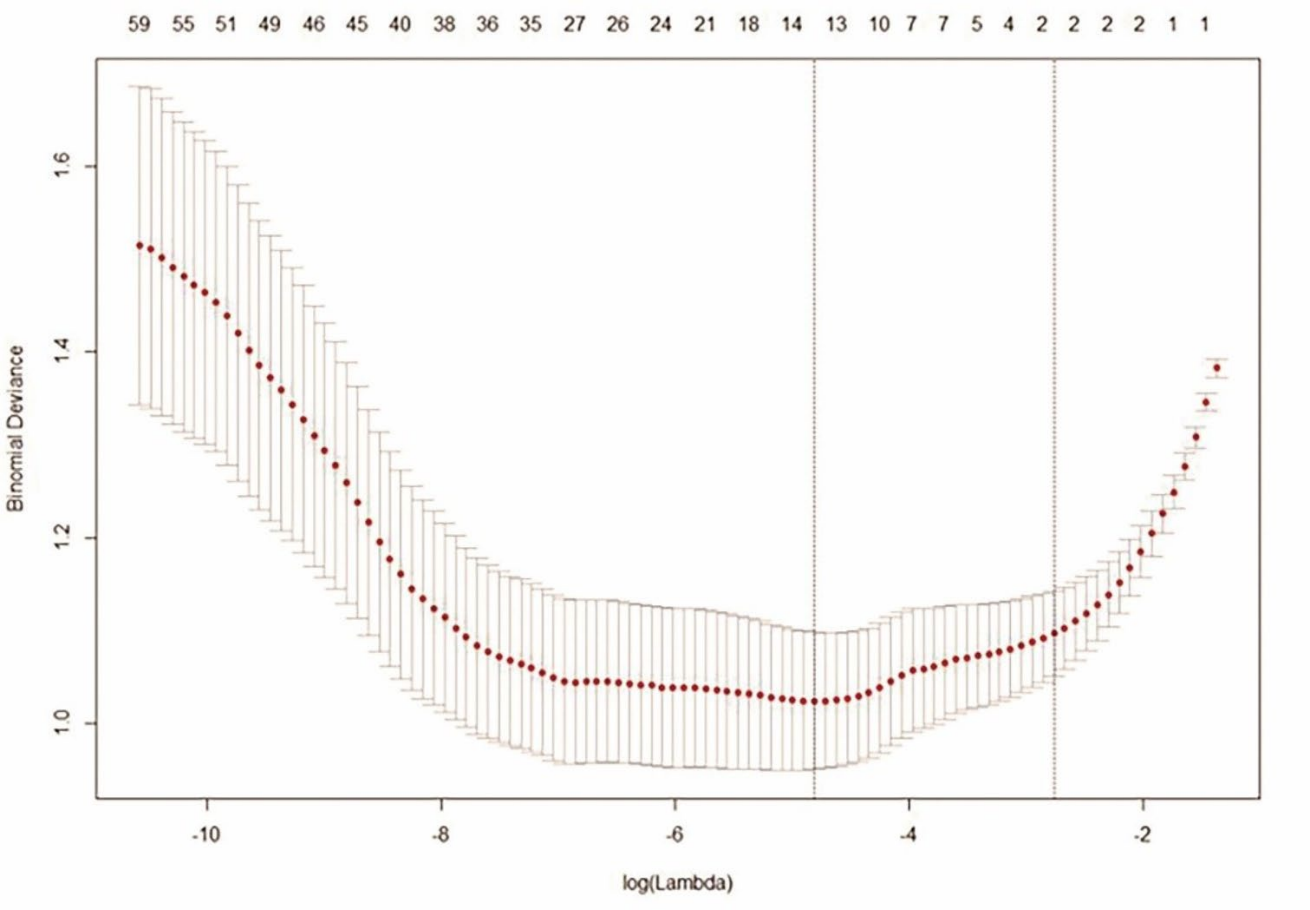
A number of radiomics features remained upon Kruskal-Wallis test and LASSO regression

A total of 82 radiomics features, with ICC > 0.75 for both intra- and inter-observer reproducibility, were selected for further analysis. A number of radiomics features (n = 14) remained upon Kruskal-Wallis test and LASSO regression (Fig. 2), while 8 features were retained after multivariate logistic regression analysis, using backward stepwise elimination method. This latest approach was used to build a Rad-score via a linear combination weight with respective coefficient (Appendix 3). The formula utilized was as it follows:

Radscore = 8.90*GLCMEnergy_angle0_offset1+-4.57*GLCMEntropy_AllDirection_offset7 + 1.01*HaralickCorrelation_angle0_offset1 + 6.41*differenceEntropy +-3.53*LowGreyLevelRunEmphasis_angle0_offset1 + 9.82*RunLengthNonuniformity_AllDirection_offset1_SD+-3.22*Compactness2 + 3.20*Sphericity+-9.05.

### Validation of the Radiomics score

**Fig. 3:**
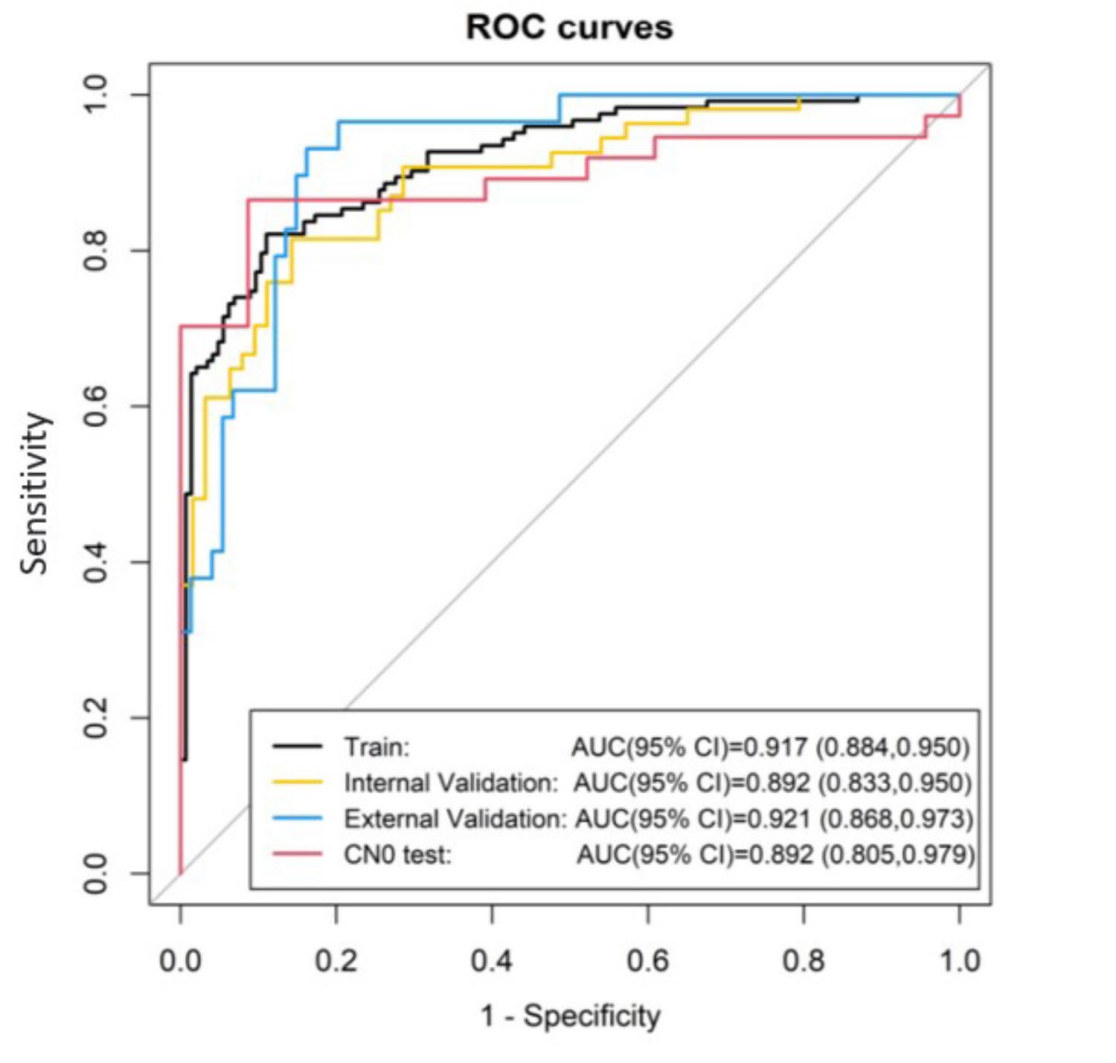
The radiomics score showed good performance to discriminate LN metastasis with AUC of 0.917(95% CI, 0.884 to 0.950), 0.892 (95% CI, 0.833 to 0.950) and 0.921 (95% CI, 868 to 0.973) in the training, internal validation cohort and external validation cohort, respectively. The test group of CN0 with a AUC of 0.892 (95% CI, 0.805 to 0.979)

The radiomics score showed good performance to discriminate LN metastasis with AUC of 0.917(95% CI, 0.884 to 0.950), 0.892 (95% CI, 0.833 to 0.950) and 0.921 (95% CI, 868 to 0.973) in the training, internal validation cohort and external validation cohort, respectively. The test group of CN0 with a AUC of 0.892 (95% CI, 0.805 to 0.979) (Fig. 3). The accuracy was 85.4% (sensitivity = 81.3%; specificity = 88.9%) in the training cohort, 82.9% (sensitivity = 79.0%; specificity = 88.7%) in the internal validation cohort, 85.4% (sensitivity = 89.7%; specificity = 83.8%) in the external validation cohort, 86.7% (sensitivity = 83.8%; specificity = 91.3%) in the CN0 test group (Table 3).

**Table 3:** The performance of radiomic score for predicting the status of LN in different datasets

Dataset	Sample Size (*N*=548)		AUC (95% CI)	Accuracy	Sensitivity	Specificity	PPV	NPV
	Positive	Negative						
Train (*N*=268)	123	145	0.917 (0.884, 0.950)	0.854	0.813	0.89	0.862	0.849
Internal Validation (*N*=117)	54	63	0.892 (0.833, 0.950)	0.829	0.79	0.889	0.854	0.812
External Validation (*N*=103)	29	74	0.921 (0.868, 0.973)	0.854	0.897	0.838	0.684	0.954
CN0_Test (*N*=60)	37	23	0.892 (0.805, 0.979)	0.867	0.838	0.913	0.939	0.778

**Fig. 4:**
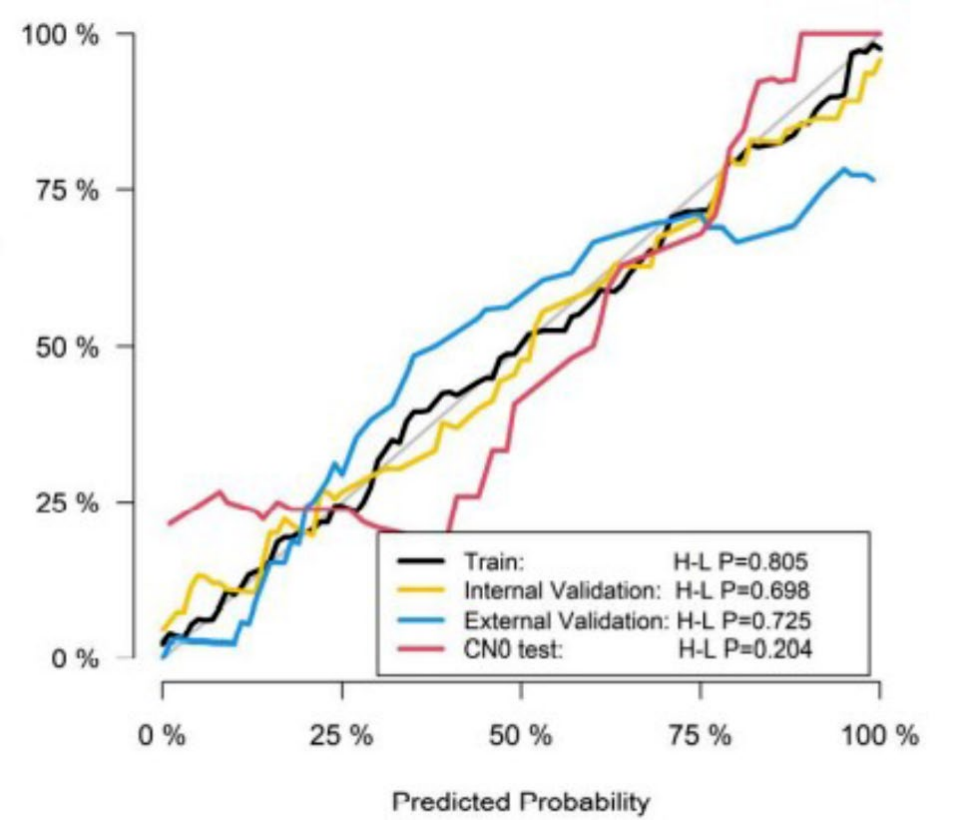
The H-L test p-value was 0.805, 0.698, 0.725 and 0.204 in training cohort, internal and external validation cohort, CN0 test group, respectively, indicating fitness of the Rad-score

**Fig. 5:**
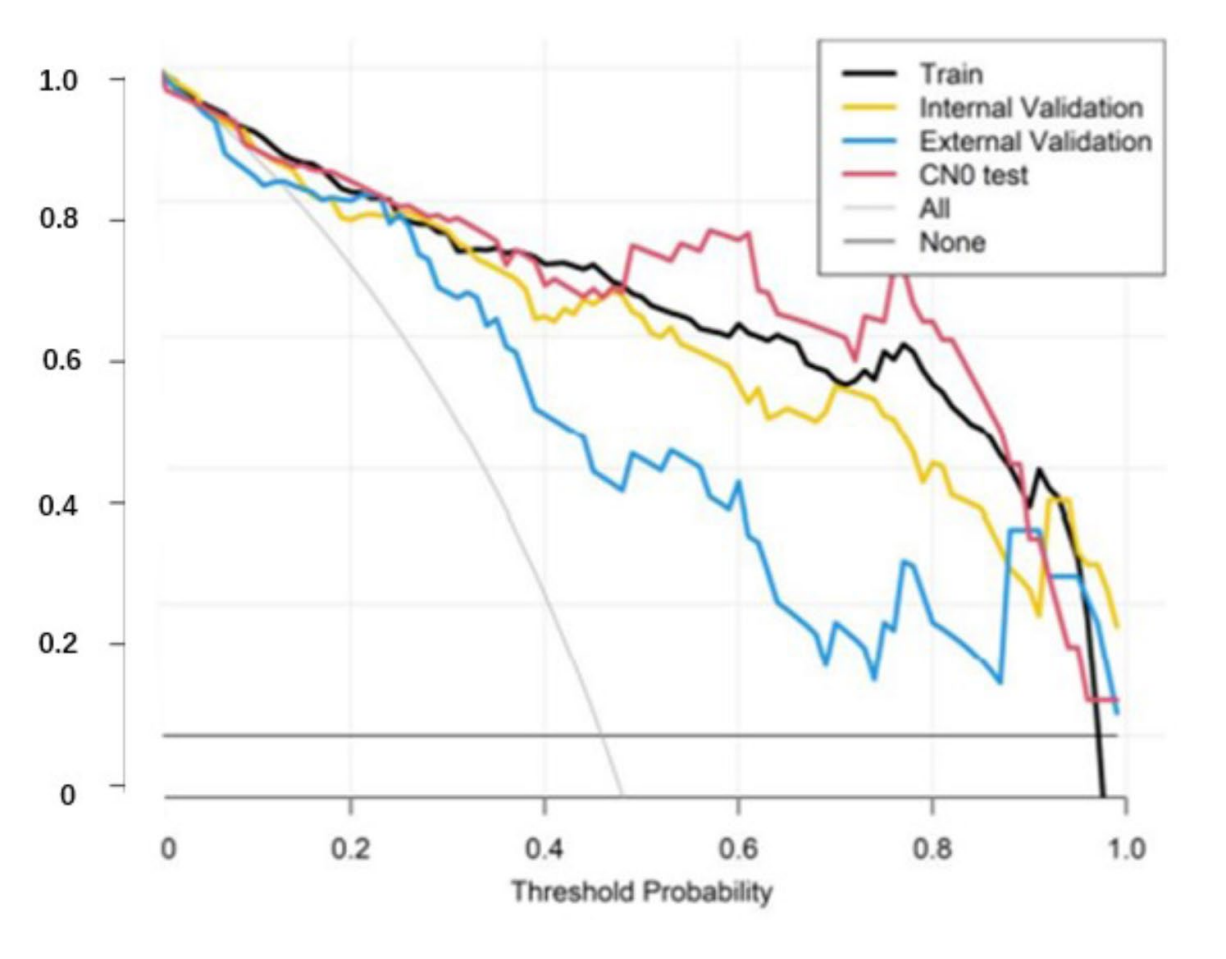
The decision curve analysis showed the clinical usefulness of the radiomics model

**Fig. 6:**
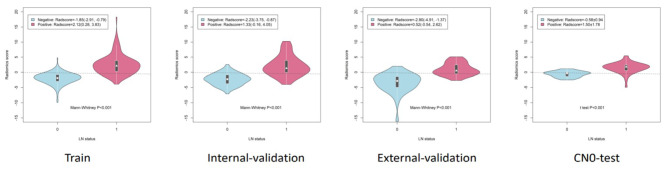
The distribution of radiomic score in different datasets

The H-L test *p*-value was 0.805,0.698,0.725 and 0.204 in training cohort, internal and external validation cohort, CN0 test group, respectively, indicating fitness of the Rad-score (Fig. 4). The decision curve analysis showed the clinical usefulness of the radiomics model (Fig. 5). The distribution of radiomic score in different datasets was shown in Fig. 6; Table 4.

**Table 4:** The distribution of radiomic score in different datasets

Dataset	Negative	Positive	Statistics	P-value
Train	-1.85(-2.91, -0.79)	2.12(0.28, 3.83)	-11.759	<0.001
Internal Validation	-2.23(-3.75, -0.87)	1.33(-0.16, 4.05)	-7.283	<0.001
External Validation	-2.80(-4.91, -1.37)	0.52(-0.54, 2.62)	-6.621	<0.001
CN0_Test	-0.58 ± 0.94	1.50 ± 1.78	-5.136	<0.001

## Discussion

Our study has presently developed and validated a CT-based radiomics model for preoperative prediction of the status of cN0 in PTC patients, using representative cohorts from two independent centers. In this context, radiomic nomogram was able to provide a potential non-invasive tool for the preoperative individualized prediction of the status of cN0 in PTC.

CT images were used to extract radiomics features to established radiomics model. Although, the most common method of cervical lymph node detection is ultrasound, however, due to the presence of sternum, US has limitations in imaging LNs in the retrosternal, and mediastinum, which restricting the detection of LNs in the central group, however, central group lymph nodes are sentinel nodes in thyroid cancer. Moreover, ultrasound diagnosis is highly dependent on personal operation, which possibly influences its reliability and reproducibility [19]. Some studys showed that CT has presented a significantly higher sensitivity and accuracy than US, but the sensitivity is still limited by 38.9–50% [12, 20–27]. Therefore, we chose to establish a CT based radiomics model. Our study have developed and validated a CT-based radiomics model with a with a balanced sensitivity (81.3%) and specificity (89.0%) in the training cohort, also have a good result in internal and external validation cohort. The results are better than Liu and colleagues’ radiomics model based on US(AUC = 0.782; ACC = 0.712) and Park and colleagues’ radiomics model based on US (AUC = 0.710 ). This indicates that our model has a good ability to predict the status of lymph nodes.

The diagnostic efficiency of the radiomics model we established is superior to other radiomics models based on CT, with AUCs of 0.917 and 0.921 in the training and external validation cohorts, a balanced sensitivity (81.3%) and specificity (89.0%) in the training cohort. Lu and colleagues established a radiomics model based on CT imaging, with the AUC of the training and validation cohorts were 0.867 and 0.822, a balanced sensitivity (72.4%) and specificity (76.3%) [28]. Zhou and colleagues established a radiomics model based on dual energy CT, with the AUC was 0.910 and 0.847 in the training and validation cohorts [29]. In their experiment, there was no restriction on the size of lymph nodes. Those lymph nodes larger than 1 cm in diameter were suggested to be metastasis by ultrasound, which indirectly increased the sensitivity of lymph node detection. In our study, we excluded lymph nodes larger than 1 cm in diameter. That is to say, the radiomics model we established had better performance in discriminating LN metastasis. Zhou and colleagues had a similar result to ours, however, it needs more data information, and the post-processing and operation process is relatively complex.

Moreover, our study predicts the CN0 and got a good result, with a AUC of 0.892 (95% CI, 0.805 to 0.979), a balanced sensitivity (83.8%) and specificity (91.3%). Although Fine-needle aspiration biopsy (FNAB)is the gold standard for the diagnosis of lymph node metastasis, one study showed FNAB had low sensitivity (11.94- 59.7%)and positive predictive value(15.69- 40.4%)for the diagnosis of indeterminate lymph nodes [30]. Our model has good detection efficiency to predict indeterminate lymph nodes, which may help to decrease the rate of unnecessary FNA for indeterminate LNs and improve the diagnosis of determinate lymph nodes. Therefore, our model can provide effective value for the diagnosis of cN0 status. This is also the innovation of our study.

In our study, the data was collected from two centers, improving the universality and reproducibility of our approach. This model was tested by a internal and external validation cohort (The external validation cohort is selected from center 2), indicating good robustness of the analysis. The CN0 test group is selected from center 1.

Nonetheless, our current study presents a few limitations. Firstly, this is a retrospective study, our data come from two centers, in which there may be some differences in CT images. Secondly, we did not incorporate clinical features to our model, since the cases we selected were too specific. Therefore, we will considered combining clinical risk factors to build the model. Our study did not include the basic CT image information into the model, because we studied clinically negative lymph nodes, and excluded those lymph nodes with obvious metastatic characteristics (maximal shortxis diameter, shape, margin, boundary, calcification, cystic change, necrosis, and enhancement, etc.).

## Conclusion

In summary, a radiomics model based on preoperative CT images of LNs was presently designed. This radiomics model, constructed according to a machine learning method, shows great diagnostic potential to preoperatively predict the status of cN0 in PTC.

### Electronic supplementary material

Below is the link to the electronic supplementary material.

Supplementary Material 1

## Data Availability

All authors have reviewed the final version of the paper and would like to take public responsibility for its content.

## Abbreviations

AUC: Area under the curve
CND: Central neck lymph node dissection
CT: Computed tomography
DCA: Decision curve analysis
H-L: Hosmer-Lemeshow
ICC: Intra/inter-class correlation coefficient
LASSO: Least absolute shrinkage and selection operator
LN: Lymph node
PTC: Papillary thyroid carcinoma
Rad-score: Radiomics score
ROC: Receiver operating characteristic
ROI: Region of interest
US: Ultrasound
CN0: Clinically node-negative
