# Correction: A CT based radiomics analysis to predict the CN0 status of thyroid papillary carcinoma: a two- center study

**DOI:** 10.1186/s40644-024-00725-4

**Published:** 2024-07-11

**Authors:** Zongbao Li, Yifan Zhong, Yan Lv, Jianzhong Zheng, Yu Hu, Yanyan Yang, Yunxi Li, Meng Sun, Siqian Liu, Yan Guo, Mengchao Zhang, Le Zhou

**Affiliations:** 1 Department of Radiology, China-Japan Union Hospital of Jilin University, Changchun, 130000 China; 2 Department of Radiology, Affiliated Fifth People’s Hospital of Chengdu University of Traditional Chinese Medicine, Chengdu, 611130 China; 3 Department of Radiology, The People’s Hospital of Bao’an, Shenzhen University, Shenzhen, 518101 China; 4 Life Sciences, GE Healthcare, Shenyang, 110000 China; 5 Department of Thyroid Surgery, China-Japan Union Hospital of Jilin University, Changchun, 130000 China

Following publication of the original article [1], we were notified that the correct affiliation of co-corresponding author Le Zhou is the Department of Thyroid Surgery, China-Japan Union Hospital of Jilin University, Changchun, 130,000, China, rather than the Department of Radiology.

The original article has been corrected.
