# Supplementary material for: A CT based radiomics analysis to predict the CN0 status of thyroid papillary carcinoma: a two- center study
Source: Cancer Imaging. 2024 May 15;24:62. doi: 10.1186/s40644-024-00690-y (PMC11094940; doi:10.1186/s40644-024-00690-y)

**Appendix 1: CT examinations**

All patients in the two hospitals underwent similar enhanced CT examinations after injection of contrast agent but with different systems and parameters. The entire neck region was covered in the CT scans. The CT image acquisition parameters of the two hospitals were shown in Table S1 as follow.

**Table S1. The CT image acquisition parameters of the two** **hospitals**

| Parameters | Hospital 1  China-Japan union hospital of Jilin university in China | Hospital 2  The People's Hospital of Bao’an in China |
| --- | --- | --- |
| CT version | Discovery CT750 HD, GE Healthcare | 64-slice spiral CT, Brilliance iCT, ROYAL PHILIPS or GE Optima CT 680 Series, GE Healthcare |
| CT tube voltage | 120 kVp | 120 kVp |
| CT tube current | tube current automatic  adjustment technology | 250 mA |
| CT rotation time | 0.60s | 0.75s |
| CT detector collimation | 64×0.625 mm | 64×0.625mm |
| Contrast agent type | Ultravist, Bayer Schering Pharma, Germany | Ultravist, Bayer Schering Pharma, Germany |
| Contrast agent concentration | 300 mgI/ml or 370 mgI/ml | 370 mgI/ml |
| Contrast agent dosage | infused 1.5 ml/kg body weight | infused 1.5 ml/kg body weight |
| Contrast agent infused rate | 4.0 ml/s | 3.0 ml/s |
| Venous phase interval time | 35 s after injection of  contrast agent | 60-70 s after injection of  contrast agent |
| Image matrix | 512×512 | 512×512 |
| Field of view | 205×205 mm | 200×200 mm |
| Reconstruction image thickness | 1.25 mm or 5 mm | 5 mm |

**Appendix 2: CT radiomics features**

A total of 396 radiomics features were extracted from lymph node CT images, including first order histogram, high order texture and morphological features. The summary of the radiomics features was shown in Table S2 as follow.

**Table** **S2.** **Summary of the radiomics features**

| Feature type | Feature name | Feature number |
| --- | --- | --- |
| 1. Histogram features | 1) Energy 2) Entropy 3) Max intensity 4) Min intensity 5) Mean value | 42 |
|  | 6) Mean absolute deviation 7) Median intensity 8) Range 9) RMS |  |
|  | 10) Standard deviation 11) Uniformity 12) Variance |  |
|  | 13) Volume Count 14) Voxel Value 15) Relative Deviation |  |
|  | 16) Frequency Size 17) Skewness 18) Kurtosis |  |
|  | 19) Quantile x (x= 0.025, 0.25, 0.5, 0.75, 0.975) |  |
|  | 20) Percentile y (y= 5, 10, 15, 20, 25, 30, 35, 40, 45, 50, 55, 60, 65, 70, 75, 80, 85, 90, 95) |  |
| 2. GLCM | 1) Energy of GLCM 2) Entropy of GLCM 3) Inertia of GLCM 4) Inverse Difference Moment | 144 |
|  | 5) Correlation 6) Cluster Prominence 7) Cluster Shade 8) Haralick Correlation |  |
|  | #Angle = All, 0, 45, 90, 135, All_SD; *Offset = 1, 4, 7 |  |
| 3. Haralick features | 1) Inverse Difference Moment 2) Angular Second Moment 3) Hara Entropy | 10 |
|  | 4) Hara Variance 5) Contrast 6) Difference Entropy 7) Difference Variance |  |
|  | 8) Sum Average 9) Sum Entropy 10) Sum Variance |  |
| 4. GLRLM | 1) Short Run Emphasis 2) Long Run Emphasis | 180 |
|  | 3) Gray Level Non-uniformity 4) Run Length Non-uniformity |  |
|  | 5) Low Gray Level Run Emphasis 6) high gray-level run emphasis |  |
|  | 7) Short Run Low Gray Level Emphasis 8) Short Run High Gray Level Emphasis |  |
|  | 9) Long Run low Gray Level Emphasis 10) Long Run High Gray Level Emphasis |  |
|  | #Angle = All, 0, 45, 90, 135, All_SD; *Offset = 1, 4, 7 |  |
| 5. Intensity Size-Zone Matrix | 1) Size Zone Variability 2) High Intensity Emphasis 3) Low Intensity Emphasis | 11 |
|  | 4) High Intensity Large Area Emphasis 5) Low Intensity Large Area Emphasis |  |
|  | 6) High Intensity Small Area Emphasis 7) Low Intensity Small Area Emphasis |  |
|  | 8) Large Area Emphasis 9) Small Area Emphasis 10) Zone Percentage |  |
|  | 11) Intensity Variability |  |
| 6. Morphological features | 1) Sphericity 2) Surface Area 3) Compactness 1 4) Compactness 2 | 9 |
|  | 5) Maximum 3D Diameter 6) Spherical Disproportion 7) Surface Volume Ratio |  |
|  | 8) Volume CC 9) Volume MM |  |
| Total radiomics features |  | 396 |

(GLCM= Grey level co-occurrence Matrix, GLRLM= Grey level run-length Matrix, CC = cm x cm x cm, MM = mm x mm x mm, 3D = three-dimensional)

**Appendix 3: The introduction and equations for the top 8 features**

The introduction and equations for the top 8 features related to cervical lymph node metastasis were as follows.

1. Energy

This feature represents the sum of squared elements in the GLCM. Range = [0 1]

Energy is 1 for a constant image. It is high when image has very good homogeneity or when pixels are very similar. The Property Energy is also known as uniformity, uniformity of energy, and angular second moment. The calculation formula is as follows:


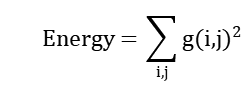


2. Entropy

Entropy is a measure of randomness of intensity image, which shows the amount of information of the image that is needed for the image compression. Entropy measures the loss of information or message in a transmitted signal and also measures the image information. The calculation formula is as follows:


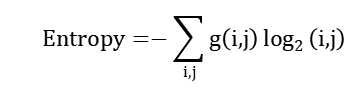


3. Haralick Correlation

This represents a measure of the linear dependency of grey levels of neighboring pixels. In other words, it measures the degree of similarity of the grey level of the image in the row or column direction. This parameter represents the local grey level correlation; the greater its value, the greater the correlation. The calculation formula is as follows:


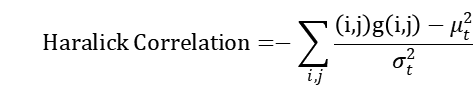


4. Difference Entropy

Difference Entropy is a measure of the randomness/variability in neighborhood intensity value differences. The calculation formula is as follows:


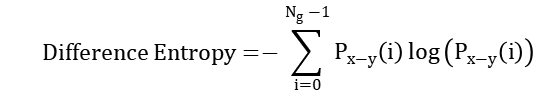


5. Run Length Non-uniformity

RLN measures the similarity of run lengths throughout the image, with a lower value indicating more homogeneity among run lengths in the image. The calculation formula is as follows:


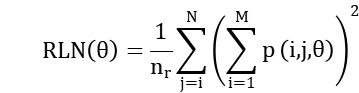


6. Low Gray Level Run Emphasis

LGRE measures the distribution of low gray-level values, with a higher value indicating a greater concentration of low gray-level values in the image. The calculation formula is as follows:


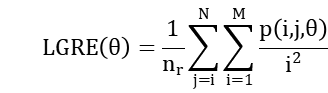


7. Sphericity

Sphericity is a measure of the roundness of the shape of the tumor region relative to a sphere. It is a dimensionless measure, independent of scale and orientation. The value range is 0<sphericity≤1, where a value of 1 indicates a perfect sphere (a sphere has the smallest possible surface area for a given volume, compared to other solids). The calculation formula is as follows:


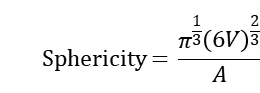


8. Compactness 2

This morphological feature describes the three-dimensional size and shape of the tumor region. In the following definitions, V denotes the volume and A, the surface area of the volume of interest. The closer this value is to 1, the closer the region of interest is to the shape of a true sphere. The calculation formula is as follows:


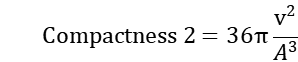

Supplement: Supplementary file 1 — Supplementary Material 1 [file 40644_2024_690_MOESM1_ESM.doc]
